# A variant in human leucine-rich repeat and coiled-coil domain-containing 1 (LRRCC1) elevates meiotic aneuploidy in oocytes

**DOI:** 10.21203/rs.3.rs-10131625/v1

**Published:** 2026-07-05

**Authors:** Marlena Duke, Karen Schindler

**Affiliations:** Rutgers, The State University of New Jersey; Rutgers, The State University of New Jersey

**Keywords:** meiosis, aneuploidy, gene variants, LRRCC1, MTOC

## Abstract

**Purpose:**

Globally, infertility rates and the age of women conceiving are both increasing. Aneuploidy is a major cause of early miscarriage, and the incidence of egg aneuploidy increases with maternal age. However, significant variation in age-related aneuploidy rates exists, suggesting that age is not the sole determinant of aneuploid conception risk. We aim to understand what variants in the human genome could predispose a woman to egg aneuploidy at an earlier than average age.

**Methods:**

The gene encoding human LRRCC1 was fused to Gfp and cloned into an oocyte expression vector designed for in vitro transcription. Site directed mutagenesis was used to create point mutations previously identified in patients with high levels of egg aneuploidy. cRNA was microinjected into mouse oocytes to observe localization, incidence of aneuploidy, and meiotic spindle parameters.

**Results:**

LRRCC1-Gfp and all variants tested localized to mouse acentrosomal microtubule organizing centers (aMTOC). Expression of the LRRCC1^H69Q^ variant elevated mouse egg aneuploidy, reduced meiosis I spindle volume and length, caused chromosome misalignment, and reduced aMTOC clustering.

**Conclusions:**

LRRCC1 promotes centrosome-independent spindle assembly during oocyte meiosis. Human genetic variants in LRRCC1, specifically p.H69Q, alter aMTOC clustering which causes abnormal spindle building, misaligned chromosomes and increased egg aneuploidy.

## Introduction

The average number of children born to a woman in her lifetime has been dropping world-wide since 1950 and is expected to reach an all-time low of 1.59 births per woman by the year 2100 [1]. This decline in fertility may be due to a number of factors such as shifting lifestyle choices or an inability to conceive or maintain a pregnancy after conception. Miscarriage, the loss of a pregnancy, is most often the result of the developing embryo or fetus possessing an incorrect number of chromosomes, or aneuploidy. The incidence of aneuploidy increases with maternal age [2]. In humans, aneuploidies are found in approximately 20% of eggs from women in their peak reproductive years (< 35 years of age) and in about 60–80% of eggs once a woman exceeds 40 years of age [3]. Many segregation errors found in aneuploid embryos are maternal in origin and most of these meiotic errors occur during meiosis I [4–6]. Although maternal age is correlated with aneuploidy, significant variation exists at each age suggesting that age is not the sole determinant [3]. Therefore, other risk factors such as environmental hazards, endocrine imbalance, lifestyle choices and genetics can influence aneuploid rates. Genetic causes are thought to underlie a significant percentage of idiopathic infertility cases [7], yet our understanding of reproductive genetics is still limited [8]. The reproductive genome therefore needs to be fully characterized to elucidate which pathogenic variants may exacerbate the age-related aneuploid risk.

We recently identified genetic variants using whole exome sequencing of over one hundred patients undergoing *in vitro* fertilization (IVF) and preimplantation genetic testing for aneuploidy (PGT-A). In that study, we compared the variants found from patients with a high (≥ 50%) and low (< 30%) proportion of aneuploid blastocysts for their given age [9]. Interestingly, patients producing a high proportion of aneuploid blastocysts carried a higher mutational burden in genes functioning in cytoskeleton and microtubule pathways [9]. Those genes included leucine-rich repeat and coiled-coil domain-containing 1 (*LRRCC1*; alt. name *CELRC*) (Table 1), centrosomal protein 120 (*CEP120*), ninein (*NIN*), pericentrin (PCNT), tubulin delta 1 (*TUBD1*), pericentriolar material 1 (*PCM1*), and HECT and RLD domain-containing E3 ubiquitin ligase 2 (*HERC2*).

LRRCC1 is a member of the evolutionarily conserved leucine-rich repeat family. It has a C-terminal coiled-coil domain and an N-terminal leucine-rich repeat domain. In HeLa and RPE1 cells, LRRCC1 localizes to pericentriolar material (PCM) of the centrosome, specifically to centriolar satellites, associating with centrosomes throughout the cell cycle and accumulating during mitosis [10–13]. LRRCC1 colocalizes with γ-tubulin in the PCM; however, LRRCC1 does not localize with spindle microtubules as γ-tubulin does [10]. Therefore, LRRCC1 localizes to PCM in a microtubule-independent manner indicating that LRRCC1 is an integral PCM component. LRRCC1 is required for the bipolar organization of mitotic spindles. siRNA-mediated LRRCC1 depletion blocked mitotic spindle formation, causing multipolar spindles, whereby centriole pairs were separated. This separation left only one centriole at each spindle pole indicating that centrosomes split into fractions in the absence of LRRCC1. Therefore, the major function of LRRCC1 during mitosis is to maintain the structural integrity of centrosomes keeping centrioles together and contribute to spindle bipolarity [10, 13].

Many centrosomal proteins and centriolar satellite proteins have been studied extensively in mitosis, yet similar structures have not been reported in oocytes. Although LRRCC1 is an integral component of the PCM and is required for the formation of normal mitotic spindles, its function in oocytes is unknown [10]. *LRRCC1* and its variants are therefore exciting candidates to deepen our understanding of how oocytes build meiotic spindles and to investigate a potential link to aneuploidy at younger than average ages. We report data that the human LRRCC1 gene variant encoding p.His69Gln elevates egg aneuploidy by impeding spindle building during meiosis I in mouse oocytes, suggesting that patients who harbor this genetic variant have a higher percentage of poor quality eggs and reduced fertility.

## Materials and Methods

### Mouse strains

CF-1 female mice (Envigo, Indianapolis, IN, USA) were used at 6–10 weeks of age for all experiments. Mice were housed in the Rutgers animal vivarium with constant temperature and 12-h light/12-h dark cycle. Food and water were provided *ad libitum*. All animals were maintained following the Rutgers Institutional Animal Care and Use Committee (IACUC) protocol #201702497 and in accordance with NIH policies.

#### Oocyte collection and in vitro maturation

Prophase I-arrested oocytes were collected from preovulatory stage follicles from the ovaries of female mice as described [14]. Mice were primed with 5 IU of pregnant mare serum gonadotrophin (PMSG) (Lee BioSolutions, Maryland Heights, MO, USA, #493 − 10) to induce follicle growth and recruitment and sacrificed 48 hours later. Ovaries were harvested and transferred to minimal essential medium (MEM) containing 2.5 μM milrinone (Sigma-Aldrich, St. Louis, MO, USA, #M4659) to prevent spontaneous meiotic resumption [15]. Follicles were pierced to release the oocytes and cumulus cells surrounding the oocytes were removed using a 100–125 μm diameter pipette. Oocytes were then transferred to microdroplets of Chatot, Ziomek and Bavister (CZB) medium under mineral oil (Sigma-Aldrich, St. Louis, MO, USA, #M5310) and cultured at 37°C in a humidified 5% CO_2_ incubator [16]. CZB was supplemented with 2.5 μM milrinone to maintain prophase I arrest during microinjection. After injection and expression of the constructs, oocytes were placed in milrinone-free CZB medium to resume meiosis and reach the desired maturation stage for evaluation: 0 h for prophase I, 3 h for early prometaphase I, 7 h for metaphase I, 11h for telophase I and 16 h for metaphase II.

### Expression constructs and cRNA synthesis

Expression constructs for cRNA synthesis were generated as previously described [17]. Briefly, the full-length coding sequence of human *LRRCC1* gene cDNA clone (Sino Biological, Wayne, PA, USA, #HG25876-U) was fused to green fluorescent protein (Gfp) to allow for protein visualization and analysis of expression levels. The *LRRCC1* clone was inserted into a pIVT-Gfp vector [18] and then transformed into *E. coli* (Takara Bio, San Jose, CA, USA, #636763). The colonies were isolated for amplification by polymerase chain reaction (PCR) followed by Sanger sequencing to select the control reference gene. Site directed mutagenesis (QuikChange Lightning Multi Site-Directed Mutagenesis Kit, Agilent, Santa Clara, CA, USA, #210515–5) was performed to generate the variant constructs for *LRRCC1* following the manufacturer’s protocol and using the following primers: *p.His69Gln* 5’-CATTGATCATATTTGGAATTTACAACAACTAGATCTGTCATCTAATCAAATAAGT-3’; *p.Leu780Pro* 5’-CACAACAAGGATCTTCTCCAGCCCAAAATCGTGGAA-3’; *p.Arg802Gln*: 5’-ATCTAGAGAGAATGAATGTCTGCAAAAGACAAATGAAAGTGATAGTG-3’. Point mutations were confirmed by Sanger sequencing. The *LRRCC1* wildtype (WT) and variant plasmids were linearized with *NdeI* restriction digest *(New England Biolabs, Ipswich, MA, USA*, #R0111S). All linearized plasmids were purified (QIAquick PCR Purification Kit, Qiagen, Aarhus, Denmark, #28104). Lastly, *in vitro* transcription (IVT) was performed (T7 mMessage mMachine Kit, Ambion, Waltham, MA, USA, #AM1340) on the purified linear double-stranded DNA templates to produce the capped cRNA for overexpression experiments.

### Microinjection

GV-intact oocytes maintained in prophase I arrest were placed in MEM medium and microinjected using a Xenoworks microinjector (Sutter Instruments, Novato, CA, USA) as previously described [19]. Oocytes were injected with 500ng/μL cRNA. Microinjected oocytes were incubated and maintained in prophase I arrest for three to four hours to allow for protein expression prior to maturation.

### Immunoblotting

Thirty mouse prometaphase I oocytes, having undergone GVBD, from each LRRCC1 overexpression group plus controls were snap frozen in liquid nitrogen after being washed extensively through protein-free MEM medium. Samples were thawed and Western Blot performed as described previously [9]. Briefly, samples were mixed with Laemmli Buffer (BioRad, Hercules, CA, USA, #1610737) and denatured at 95°C for 5 min. Proteins were separated on a 7.5% precast polyacrylamide gel (BioRad, Hercules, CA, USA, #4561023) and transferred onto a 0.2 μm polyvinylidene difluoride (PVDF) membrane (BioRad, Hercules, CA, USA, #1704156). Blocking and washing were performed in tris-buffered saline (TBS) with 0.1% Tween 20 (MilliporeSigma, Burlington, MA, USA, #274348) and 2% skim milk (Cytiva, Marlborough, MA, USA, #RPN418). The membrane was incubated with blocking solution for 1 h at room temperature, then incubated with rabbit anti-GFP primary antibody (1:500; MilliporeSigma, Burlington, MA, USA, #G1544) overnight at 4°C. The membrane was washed again in blocking solution and then incubated for 1 h with rabbit secondary antibody from the KwikQuant Western Blot Kit (1:1000; Kindle Biosciences, Greenwich, CT, USA, #R1006). After GFP detection, the membrane was treated for 1 h with Restore Plus Western Blot Stripping Buffer (Thermo Scientific, Waltham, MA, USA, #46430) to remove the antibodies prior to probing with rabbit anti-α-tubulin primary antibody (1:1000; Cell Signaling Technology, Danvers, MA, USA, #2125) for normalization. Protein bands were detected using the KwikQuant Western Blot Detection Kit (Kindle Biosciences, Greenwich, CT, USA, #R1002).

### Immunofluorescence

Immunofluorescence was performed as described [14]. Oocytes were fixed in 2% paraformaldehyde (PFA; MilliporeSigma, Burlington, MA, USA, #P6148) in phosphate-buffered saline (PBS) for 20 min at room temperature. The fixative was washed out through three drops of blocking buffer (0.3% BSA and 0.1% Tween-20 in PBS) incubating for 10 min each. The oocytes were permeabilized in PBS containing 0.2% Triton X-100 for 20 min followed by three 10 min washes in blocking buffer. Immunostaining was performed by incubating oocytes with primary antibodies for 1 h and secondary antibodies for 1 h in a dark humidified chamber at room temperature. Following each primary and secondary incubation, three 10 min washes in blocking buffer were performed. Finally, the oocytes were mounted in VectaShield (Vector Laboratories, Newark, CA, USA, #H-1000) containing 4’, 6- Diamidino-2-Phenylindole, Dihydrochloride (DAPI; Life Technologies, Carlsbad, CA, USA, #D1306; 1:170).

The following primary antibodies were used for immunostaining: human anti-centromeric antigen (1:30; Antibodies Incorporated, Davis, CA, USA, #15–234), mouse anti-pericentrin (PCNT) (1:100; BD Biosciences, San Jose, CA, USA, #611814), sheep anti-α/β-tubulin (1:100; Cytoskeleton, Denver, CO, USA, #ATN02). The following secondary antibodies (1:200) were used: anti-mouse-Alexa-488 (Life Technologies, Carlsbad, CA, USA, #A11029), anti-sheep-Alexa-568 (Life Technologies, Carlsbad, CA, USA, #A21099), and anti-human-Alexa-633 (Life Technologies, Carlsbad, CA, USA, #A21091).

#### In situ chromosome counting

To assess chromosome number, intact metaphase II eggs were treated with monastrol as previously described [19]. Eggs were incubated for at least 2 h in CZB with 100 μM monastrol (MilliporeSigma, Burlington, MA, USA, #M8515) dissolved in dimethyl sulfoxide (DMSO; MilliporeSigma, Burlington, MA, USA, #472301). The eggs were fixed and stained with anti-centromeric antibody to label kinetochores and mounted in Vectashield containing DAPI to label the chromosomes. The kinetochores were counted by analyzing serial 0.5 μm confocal sections and rotating the 3D image using Bitplane Imaris. Mouse eggs were euploid when containing 20 pairs of sister chromatids (40 kinetochores) and were aneuploid when deviating from this number.

### Image analysis

Confocal image z-slices were projected in 2D or reconstructed into a 3D model for analysis using NIH ImageJ software or Bitplane Imaris software. Bipolar spindle length was measured between the farthest points of the spindle ends using the line tool. Spindle and MTOC volume were assessed with the surface detection function and pixel intensity was assessed as the average within the surface. Metaphase plate width (chromosome alignment) was measured between the two farthest chromosome homologs on either side of the spindle midzone using the line tool and normalized to the length of the spindle. Spindle measurements were normalized to the WT mean for each replicate and relative results compared.

### Statistical analysis

All experiments were performed at least three times unless otherwise noted. Student T-test or one-way ANOVA were used to evaluate significant differences between groups using GraphPad Prism software. Error bars indicate the standard error of the mean (SEM) or standard deviation (SD).

## Results

### LRRCC1 localizes to aMTOCs in mouse oocytes

In a previous study, we identified patients who harbored genetic variants in the LRRCC1 gene that functions at spindle poles in mitotic cells (Table 1) [9]. In that study we chose to focus on CEP120, because it was the most significantly burdened gene in the data set and we found that genetic variation in CEP120 altered spindle building and caused elevated rates of meiotic aneuploidy. The presence and function of LRRCC1 in oocytes was unknown. To determine if *LRRCC1* variation can elevate egg aneuploidy, we pursued further analysis of this gene and the three identified variants using mouse oocytes (Table 1). We elected to use mouse oocytes for functional analyses because, not only is the process of meiosis conserved between human and mouse, but we can control genetic background and statistically power the study by significant oocyte numbers, both of which are not possible when using human oocytes.

**Figure 1:**
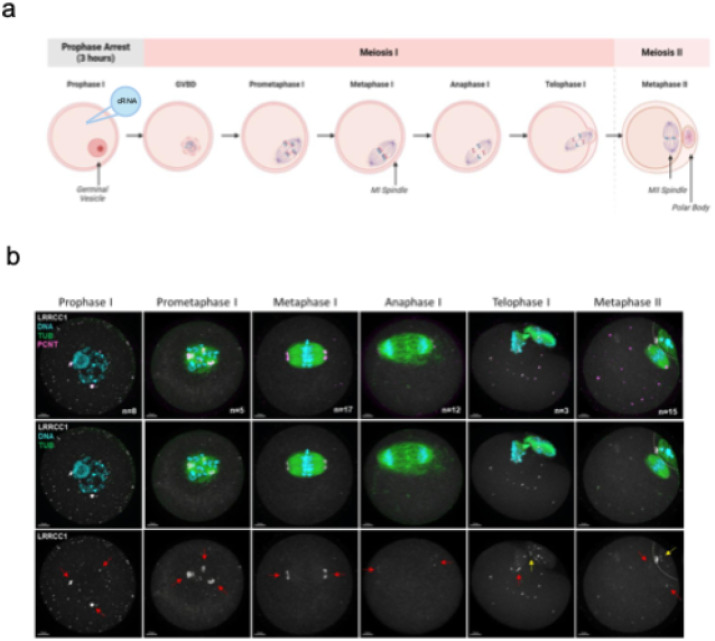
Expression of exogenous LRRCC1 protein. a) Schematic of workflow and oocyte meiotic maturation. Image created by BioRender. b) Confocal images of LRRCC1-Gfp expression in mouse oocytes (GFP; gray), DNA (DAPI; blue), spindle (α-tubulin; green) and aMTOCs (pericentrin, PCNT; magenta). Red arrows indicate LRRCC1 localization. Yellow arrows indicate LRRCC1 localization in polar body. n, total number oocytes examined for each meiotic stage in 2 experimental replicates. Scale bar: 10μm.

We first attempted to determine the expression and localization of mouse LRRCC1. However, commercially available antibodies failed to specifically detect the protein, despite evidence indicating that *Lrrcc1* is a maternally expressed gene [20]. As alternative approach to assess LRRCC1 localization in mouse oocytes, we prepared a *Gfp*-tagged human *LRRCC1* construct for microinjection of *in vitro* transcribed RNA into prophase I-arrested oocytes. Injected oocytes were maintained in prophase I arrest for 3 hours to allow for translation and then, once released from arrest, fixed at various stages of meiosis I and II (Fig. 1a). LRRCC1-GFP localized to aMTOCs as determined by co-localization with the aMTOC backbone protein PCNT (Fig. 1b). Co-localization with PCNT occurred at all meiotic stages examined. Localization of LRRCC1 in oocytes at aMTOCs is consistent with its localization at MTOCs in somatic cells [10]. Of note, LRRCC1-Gfp also localized in the first polar body and in the midbody region between the oocyte and polar body [21].

### LRRCC1 gene variants localize to aMTOCs

**Figure 2:**
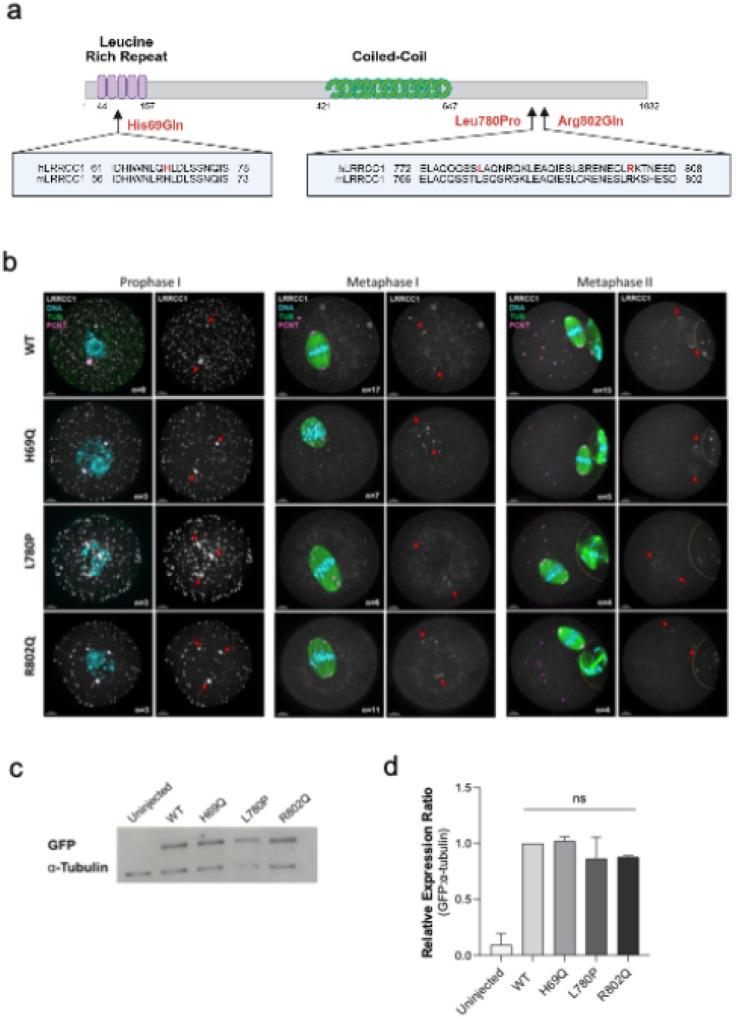
Localization of LRRCC1-Gfp variants does not differ from WT. **(a)** Schematic of LRRCC1 protein with conserved domains and variants labeled. h is human protein sequence and m is mouse protein sequence. Image created by BioRender. **(b)** Confocal images of LRRCC1 variant localization during meiotic maturation: GFP-LRRCC1 (WT), GFP-LRRCC1^H69Q^ (H69Q), GFP-LRRCC1^L780P^ (L780P), and GFP-LRRCC1^R802Q^ (R802Q). In overlay, LRRCC1 (GFP; gray), DNA (DAPI; blue), spindle (α-tubulin; green) and aMTOC (PCNT; magenta). Red arrows indicate LRRCC1 localization. n, total number oocytes per meiotic stage examined in 2 experimental replicates. Scale bar: 10μm. **(c)** Western blot detecting GFP in uninjected control oocytes and oocytes injected with indicated constructs. α-Tubulin served as loading control. 30 oocytes per group. **(d)** Quantification of relative GFP/α-tubulin ratio. Bars and error bars indicate ± SEM. One-way ANOVA used to determine significance. ns, not significant. Full length blot is Figure S1.

We previously identified three genetic variants in patients with elevated levels of blastocyst aneuploidy (Table 1) [9]. One variant changes Histidine 69 to a glutamine and is in the leucine rich repeat region of LRRCC1 (Fig. 2a). The other two variants are located in the C-terminal region and change leucine 780 to a proline and arginine 802 to a glutamine (Fig. 2a). Having established that LRRCC1 localizes to aMTOCs throughout meiosis, we asked if localization differs between the wildtype (WT) and variant proteins. *Gfp-*tagged LRRCC1 cRNA encoding each variant (*LRRCC1, p.His69Gln, p.Leu780Pro, p.Arg802Gln*) were injected into prophase I mouse oocytes and allowed 3 hours for translation (Fig. 1a). Upon expression in WT oocytes, LRRCC1-Gfp and each LRRCC1 variant localized similarly to the aMTOCs surrounding the nuclear envelope in prophase I-arrested oocytes, and to the aMTOCs at meiotic spindle poles in metaphase I and metaphase II (Fig. 2b). To confirm that the injected WT and variant constructs were expressed to similar levels, we performed western blot analysis (Fig. 2c) to compare the intensities of GFP relative to spindle α-tubulin, as protein loading control (Fig. 2c–d). Importantly, there was no difference in expression levels between WT and LRRCC1 variants. These data indicate that the variants do not alter protein stability or localization.

### LRRCC1 variants affect chromosome segregation

**Figure 3:**
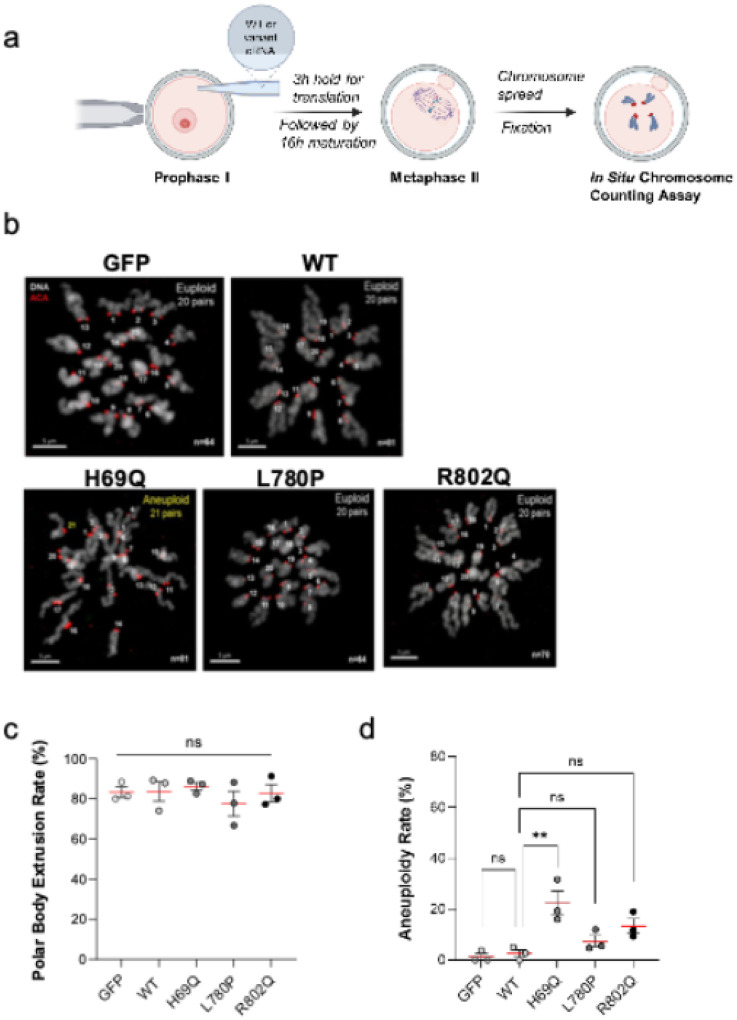
LRRCC1^H69Q^ variant causes increased egg aneuploidy. **(a)** Schematic of experimental design to assess oocyte maturation and aneuploidy. Image created by BioRender. **(b)** Confocal images from *in situ* chromosome counting assay on metaphase II eggs expressing the indicated LRRCC1 constructs. Eggs were stained to detect centromeres (anti-centromeric antigen, ACA; red) and DNA (DAPI; gray). Extra chromosome indicated in yellow. n, total number oocytes examined in 3 experimental replicates. Scale bar: 5μm. **(c-d)** Frequency of polar body extrusion and aneuploidy in oocytes expressing GFP control and LRRCC1 WT and variants as indicated. One-way ANOVA used to determine significance. Bars and error bars indicate ± SEM. ns, not significant; **P<0.01.

Because we previously identified LRRCC1 variants in patients with high rates of aneuploid conception [9], we asked whether expression of these variants would alter the rate of egg aneuploidy in otherwise WT oocytes. Prophase I oocytes were injected with a control GFP cRNA, WT LRRCC1 or variant LRRCC1 cRNA and cultured to metaphase II. Chromosomes were then spread through incubation with monastrol, a kinesin Eg5 inhibitor which collapses the bipolar spindle into a monopolar spindle, enabling ease of counting chromosomes (Fig. 3a–b) [19]. LRRCC1 variants did not interfere with oocyte cell cycle progression because a similar rate of oocytes reached metaphase II, as determined by rates of first polar body extrusion between the control and variant-expressing groups (Fig. 3c). Interestingly, expression of the LRRCC1^H69Q^ variant caused a significant increase in the proportion of aneuploid egg production compared to WT LRRCC1 (Fig. 3d) (2% vs 24%). The rate of aneuploid eggs after expression of the LRRCC1^R802Q^ variant showed a trend (p < 0.1) towards an increase compared to the WT. Therefore, we elected to further pursue both the LRRCC1^H69Q^ and LRRCC1^R802Q^ variants in understanding how they disrupt chromosome segregation. Because expression of the LRRCC1^L780P^ variant resulted in a similar rate of aneuploidy compared to WT, we eliminated this variant from further examination.

### LRRCC1^H69Q^ variant alters meiotic MI spindle morphology

**Figure 4:**
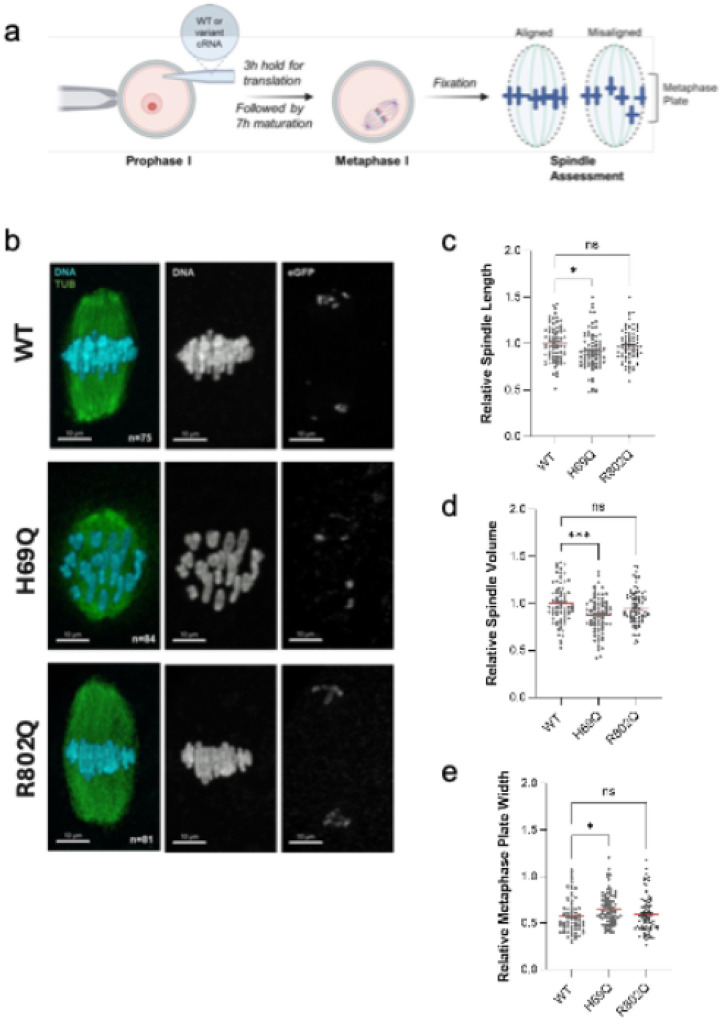
LRRCC1^H69Q^ variant causes smaller MI spindles and misaligned chromosomes. **(a)** Schematic of experimental design to assess MI spindle phenotypes. Image created by BioRender. **(b)** Confocal images of MI oocytes expressing the indicated LRRCC1 constructs stained to detect the spindle (α-tubulin; green), DNA (DAPI; blue) and LRRCC1 (GFP; gray). n, total number oocytes examined in 3 experimenal replicates. Scale bar: 10 μm. **(c-d)** Quantification of the relative MI spindle length and spindle volume. **(e)** Quantification of the relative MI metaphase plate width signifying chromosome alignment. Mean indicated ± SD. One-way ANOVA used to determine significance. ns, not significant; *P < 0.05, ***P < 0.001.

Because many chromosome segregation errors found in aneuploid embryos are maternal in origin and occur during meiosis I [4–6], and because LRRCC1 localizes to meiotic spindle poles (Fig. 1b), we next examined potential defects in MI spindle morphology. Prophase I oocytes were injected with WT or variant LRRCC1 cRNA and cultured to metaphase I to assess spindle parameters (Fig. 4a–b). Expression of the LRRCC1^H69Q^ variant resulted in MI spindles that were shorter in length and smaller in volume relative to WT LRRCC1-expressing oocytes (Fig. 4b–d). Additionally, expression of LRRCC1^H69Q^ resulted in an increased incidence of chromosome misalignment represented by a greater metaphase I plate width relative to WT-expressing oocytes (Fig. 4e). By contrast, oocytes expressing the LRRCC1^R802Q^ variant showed no variation in spindle morphology relative to oocytes expressing WT LRRCC1. The abnormal LRRCC1^H69Q^ MI spindle phenotype suggests that meiotic MI spindle assembly is altered, and that overexpression of the variant protein interferes with microtubule nucleation from aMTOCs.

### LRRCC1^H69Q^ variant oocytes have altered aMTOC clustering

**Figure 5:**
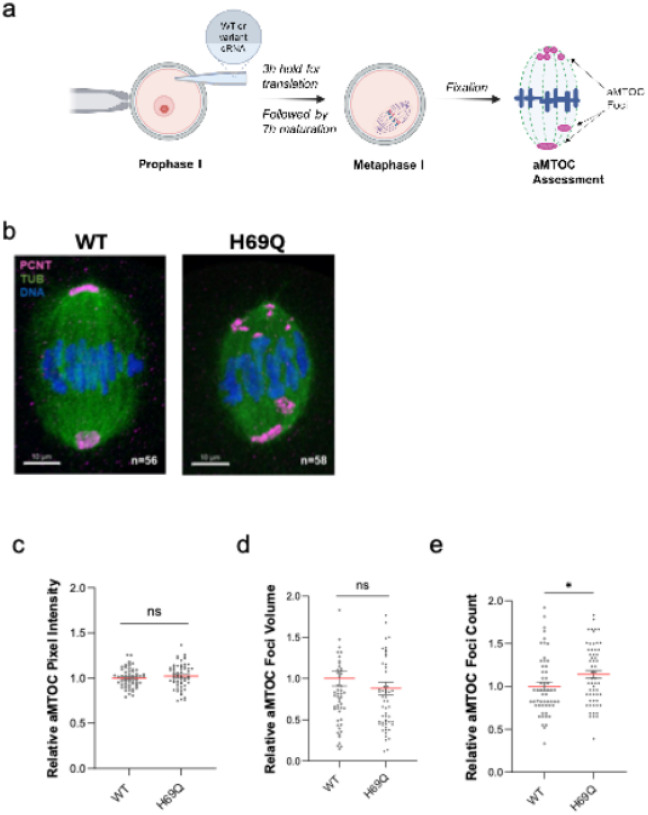
LRRCC1^H69Q^ variant alters aMTOC clustering. **(a)** Experimental design to assess MI spindle aMTOC phenotype. Image created by BioRender. **(b)** Confocal images of MI oocytes expressing the indicated LRRCC1 constructs stained to detect the aMTOCs (PCNT; magenta), spindle (α-tubulin; green) and DNA (DAPI; blue). n, total number oocytes examined in 3 experimental replicates. Scale bar: 10 μm. **(c-e)** Quantification of the relative metaphase I spindle aMTOC pixel intensity, relative foci volume and relative foci count. Mean indicated ± SD. Student’s t-test used to determine significance. ns, not significant. *P < 0.05.

Because spindle morphology was altered upon LRRCC1^H69Q^ expression and LRRCC1 localizes to aMTOCs, we next evaluated aMTOC morphology. Prophase I oocytes were injected with WT LRRCC1 or LRRCC1^H69Q^ variant cRNA and cultured to metaphase I to assess aMTOC morphology using PCNT as the aMTOC marker (Fig. 5a–b). Expression of LRRCC1^H69Q^ did not alter the relative intensity of PCNT at each aMTOC foci (Fig. 5c) indicating PCNT recruitment to or incorporation in aMTOCs were not affected. Additionally, LRRCC1^H69Q^ expression did not alter the relative volume of aMTOC foci in MI oocytes (Fig. 5d). However, the number of aMTOC foci in MI oocytes was increased when LRRCC1^H69Q^ was expressed compared to control injected oocytes (Fig. 5e). It appeared that aMTOC fragmentation and distribution were appropriately achieved, yet the final step of aMTOC clustering was delayed or deficient compared to control oocytes thus interfering with the timely assembly of a normal bipolar spindle with proper chromosome alignment. This abnormal clustering did not interfere with the ability of the variant oocytes to ultimately progress to metaphase II and first polar body extrusion (Fig. 3c); however, it did interfere with proper chromosome segregation (Fig. 3d).

## Discussion

Using an *in vitro* mouse oocyte system, we report that LRRCC1 localizes to aMTOCs at all meiotic stages investigated. When the three variants of LRRCC1 were evaluated for pathogenicity, there was no change in protein localization. Importantly, the LRRCC1^H69Q^ variant altered MI spindle building, causing misaligned chromosomes and increased egg aneuploidy. Upon closer assessment, LRRCC1^H69Q^ overexpression appeared to alter aMTOC clustering in the MI spindle, which may be the cause or result of delayed spindle building that ultimately interfered with correct chromosome segregation.

Because a nonsynonymous point mutation that changes the amino acid sequence in a protein domain could disrupt the protein function due to a modification in structure, stability, or interaction with other proteins or molecules, it is possible that the histidine to glutamine substitution alters these critical functions. Histidine 69 is located in the leucine-rich repeat (LRR) domain at the N-terminus of the protein (Fig. 2a). LRR domains are conserved sequence motifs that serve as a structural framework for protein-protein interactions [22]. Histidine and glutamine have hydrophilic side chains; however, their side chain properties differ because histidine has a positively charged imidazole ring at physiological pH, while glutamine is uncharged and can form hydrogen bonds. Because LRR domains have specific structural features based on repeating leucine residues, a single amino acid change could disrupt the binding specificity and weaken the interaction with its target protein.

Although the function of LRRCC1 in oocytes had not yet been investigated, it is required for the formation of mitotic spindles [10]. LRRCC1 is a component of the centriolar satellite and centrosomal interactome and there is a substantial overlap between these two groups of proteins [23]. Centriolar satellites were named for their location surrounding, but separate from, centrioles. Centriolar satellites are ubiquitous membrane-less organelles that possess a liquid-like phase separation ability to adapt their physical and compositional properties in changing conditions [23]. The primary function of centriolar satellites is to modulate the levels of proteins at the centrosome through two distinct mechanisms. First, centriolar satellites act as sequestration sites for proteins, kinases, enzymes and deubiquitinating (DUB) agents and regulate their availability to the centrosome [23, 24]. By spatially concentrating enzymes with their substrates, it is suggested that satellites are regulators of proteostasis by controlling the turnover of resident proteins. Second, a subset of centriolar satellites transports specific proteins to the centrosomes and moves them along microtubules, away from or towards the centrosome, using the microtubule motor dynein [25]. PCM1 is the scaffolding backbone of the centriolar satellites and facilitates the recruitment of proteins including pericentrin, the scaffolding backbone of the centrosome [26, 27]. LRRCC1 and PCM1 are binding partners and therefore it is likely that LRRCC1 is either couriered by PCM1 or involved in couriering of centrosomal proteins necessary for microtubule nucleation [12, 28–30].

In mouse oocytes, LRRCC1^H69Q^ overexpression altered aMTOC clustering in the MI spindle. This clustering defect may be the cause or result of delayed spindle building. The clustering of aMTOCs during meiotic metaphase I in mouse oocytes is also controlled by Aurora kinase A (AURKA) activity [31]. AURKA is a member of the evolutionarily conserved Aurora serine/threonine kinase family and a pleiotropic regulator of meiotic maturation [32]. A decrease in AURKA activity results in many smaller foci of PCNT and CEP215 mislocalization at spindle poles instead of the typical ring-like structure exhibited from normal aMTOC clustering [31]. AURKA localization, abundance and activity are tightly regulated by centriolar satellites to ensure that it functions at the right time and place [24, 33]. AURKA can specifically interact with PCM1 at the satellites [24]. Therefore, it is possible that the H69Q variant, a centriolar satellite protein that binds to PCM1, interferes with AURKA activity through altering the sequestration or limiting the transport of AURKA or its activators to the centrosome, or negatively regulating AURKA stability to ultimately interfere with normal aMTOC clustering.

Evaluating human gene variants through overexpression in mouse oocytes has a few limitations. First, expression of the injected variants could compete with endogenous protein causing a varied result depending on the level of expression and sensitivity of each oocyte. Second, overexpression leads to protein levels that supraphysiological which could disrupt the balance of protein complexes or overwhelm cellular pathways, leading to unintended consequences [34]. Both of these concerns are mitigated by expressing WT controls and by expressing benign variants. Finally, there are differences between mouse and human meiotic spindle building [5, 35] and it is possible that the variant may affect mouse oocytes differently than human oocytes.

LRRCC1 has not yet been investigated in human oocytes. In mouse oocytes, LRRCC1 localizes to aMTOCs, similar to centrosomal proteins PCNT and CEP192. In human oocytes that do not have polar aMTOCs, PCNT is absent; meanwhile, CEP192 localizes to microtubules. The centriolar satellite proteins PCM1 and TACC3 that form the liquid-like spindle domain (LISD) localize to the spindle poles and microtubules in mouse oocytes and localize to the microtubules in human oocytes [36, 37]. LRRCC1 variants were identified through algorithms as suspected of having a functional impact on meiotic spindle building and this concern was confirmed for the LRRCC1^H69Q^ variant in an *in vitro* mouse validation model. At this time, it is difficult to predict how the H69Q variant would alter LRRCC1 function if present in human oocytes; however, based on the variant impact in mouse oocytes, it is worth continuing the investigation as a potential cause of human egg aneuploidy at younger than average ages.

## Conclusion

In summary, these data suggest that LRRCC1 colocalizes with mouse oocyte aMTOCs and is another important PCM protein promoting centrosome-independent spindle assembly during meiosis. In these experiments, the LRRCC1^H69Q^ variant appeared to interfere with normal aMTOC clustering causing delayed or abnormal spindle building and ultimately resulting in misaligned chromosomes and increased egg aneuploidy.

## Supplementary Files

This is a list of supplementary files associated with this preprint. Click to download.

- FigureS1.pdf

## Figures and Tables

**Table 1:** Whole exome sequencing and gene prioritization identified LRRCC1 candidate gene with variants linked to spindle building.

Gene	Variant	dbSNP ID	Amino acid change	Functional consequence	Allele frequency
*LRRCC1*	NM_033402.4:c207T > A	rs16913589	p.His69Gln	Deleterious/Possibly damaging	4.26×10^−2^
*LRRCC1*	NM_033402.4:2339T > C	rs147274148	p.Leu780Pro	Deleterious/Possibly damaging	5.90×10^−3^
*LRRCC1*	NM_033402.4:c2405G > A	rs779167117	p.Arg802Gln	Deleterious/Possibly damaging	6.39×10^−6^

LRRCC1, leucine-rich repeat and coiled-coil centrosomal protein 1. Predicted functional consequences are from SIFT and Polyphen 2 and allele frequencies are from gnomAD v4.1.1. Data adapted from [9].

## Data Availability

Images and constructs will be available upon request.
